# Rosai-Dorfman Disease and Unusual Local Invasive Presentation

**DOI:** 10.7759/cureus.7328

**Published:** 2020-03-19

**Authors:** Oranus Mohammadi, Miriam Zylberglait Lisigurski, Divy Mehra, Reza Pishdad, Seza Gulec

**Affiliations:** 1 Internal Medicine, Aventura Hospital and Medical Center, Aventura, USA; 2 Ophthalmology, Nova Southeastern University Dr. Kiran C. Patel College of Osteopathic Medicine, Miami, USA; 3 Internal Medicine, Rutgers New Jersey Medical School, Newark, USA; 4 Surgical Oncology, Herbert Wertheim College of Medicine, Florida International University, Miami, USA

**Keywords:** rosai dorfman disease, sinus histiocytosis with massive lymphadenopathy

## Abstract

Rosai-Dorfman disease (RDD) is a rare medical condition with bilateral painless lymphadenopathy. We present the case of a young man diagnosed with a very unique presentation of Rosai-Dorfman disease.

A 40-year-old African-American man presented with a firm, non-tender, progressive chest and neck mass appeared three months ago. Imaging of the neck demonstrated an 8.6-cm anterior neck subcutaneous soft tissue mass extending into the anterior mediastinum through the sternum with erosive changes in the sternum and the lesion is abutting the right common carotid artery and innominate vein and surrounds the medial aspect of the clavicles bilaterally. Ultrasound (US)-guided biopsy showed marked polytypic-appearing plasma cell proliferation associated with relatively prominent histiocytes with hemophagocytosis/emperipolesis and focal neutrophils. There were S100+ histiocytes; however, findings were not typical for RDD. As that biopsy was not diagnostic, incisional biopsy with adequate sampling was performed. Surgical pathology demonstrated a very abnormal infiltrate with prominent histiocytes including areas with the features of extranodal RDD. BRAF V600E immunohistochemistry (IHC) was negative. Modified radical neck dissection, proximal sternal resection and superior mediastinal nodal dissection surgery was recommended. However, the patient refused the procedure.

Typical manifestations are lymphadenopathy with fever that our patient did not experience. Bone involvement happens in 5-10% of cases. There is not enough data about blood vessel invasion which make our case unique. Treatment plan is still controversial. Clinical monitoring is recommended if the symptoms are tolerable as regression has been reported in many cases (20-50%). Surgery is reserved for patients with vital organ involvement or extra-nodal disease.

## Introduction

Rosai-Dorfman disease (RDD) is a rare benign medical condition that primarily manifests with bilateral painless lymphadenopathy, fever, and neutrophilia in adolescents and young adults. Concomitant lymphadenopathy with extranodal involvement occurs in 43% of cases. Isolated extranodal involvement is reported in 23% of patients, most commonly in the area of the head and neck [1]. Invasion of the major vessels is exceedingly rare. While RDD is usually benign, aggressive forms have been documented in the literature [2]. We present the case of a young man diagnosed with a unique presentation of Rosai-Dorfman disease.

## Case presentation

**Figure 1:**
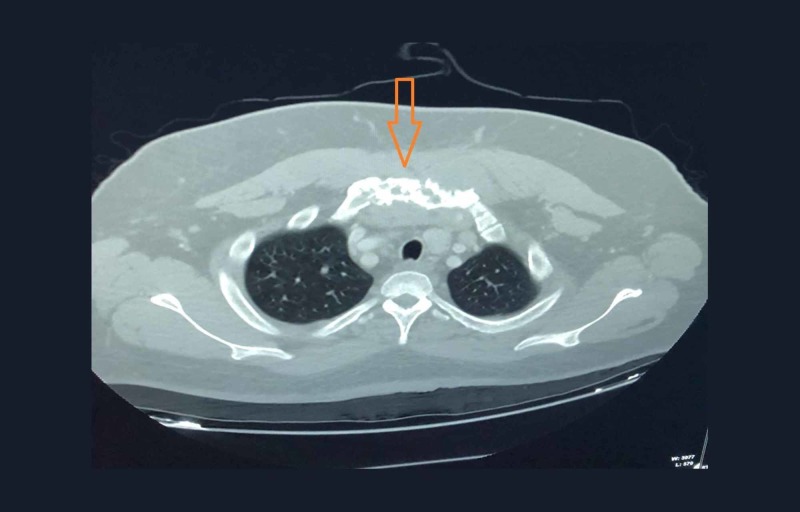
CT scan of the neck demonstrates an 8.6-cm anterior neck subcutaneous soft tissue mass extending into the anterior mediastinum through the sternum, with erosive changes visible in the sternum. The lesion is adjacent to the right common carotid artery and innominate vein, and surrounds the medial aspect of the clavicles bilaterally.

A 40-year-old African-American man presented with a firm, non-tender, upper chest and neck mass of sudden-onset three months ago that had been slowly growing since. He denied difficulty swallowing, vocal hoarseness, neck or chest pain and shortness of breath. He reported intentional weight loss of 30 lbs over the past three months. Computed tomography (CT) scan of the anterior neck demonstrated an 8.6-cm subcutaneous soft tissue mass extending into the anterior mediastinum through the sternum, which demonstrated erosive changes (Figure 1). The lesion was visible abutting the right common carotid artery and innominate vein, surrounding the medial aspect of the clavicles bilaterally.

**Figure 2:**
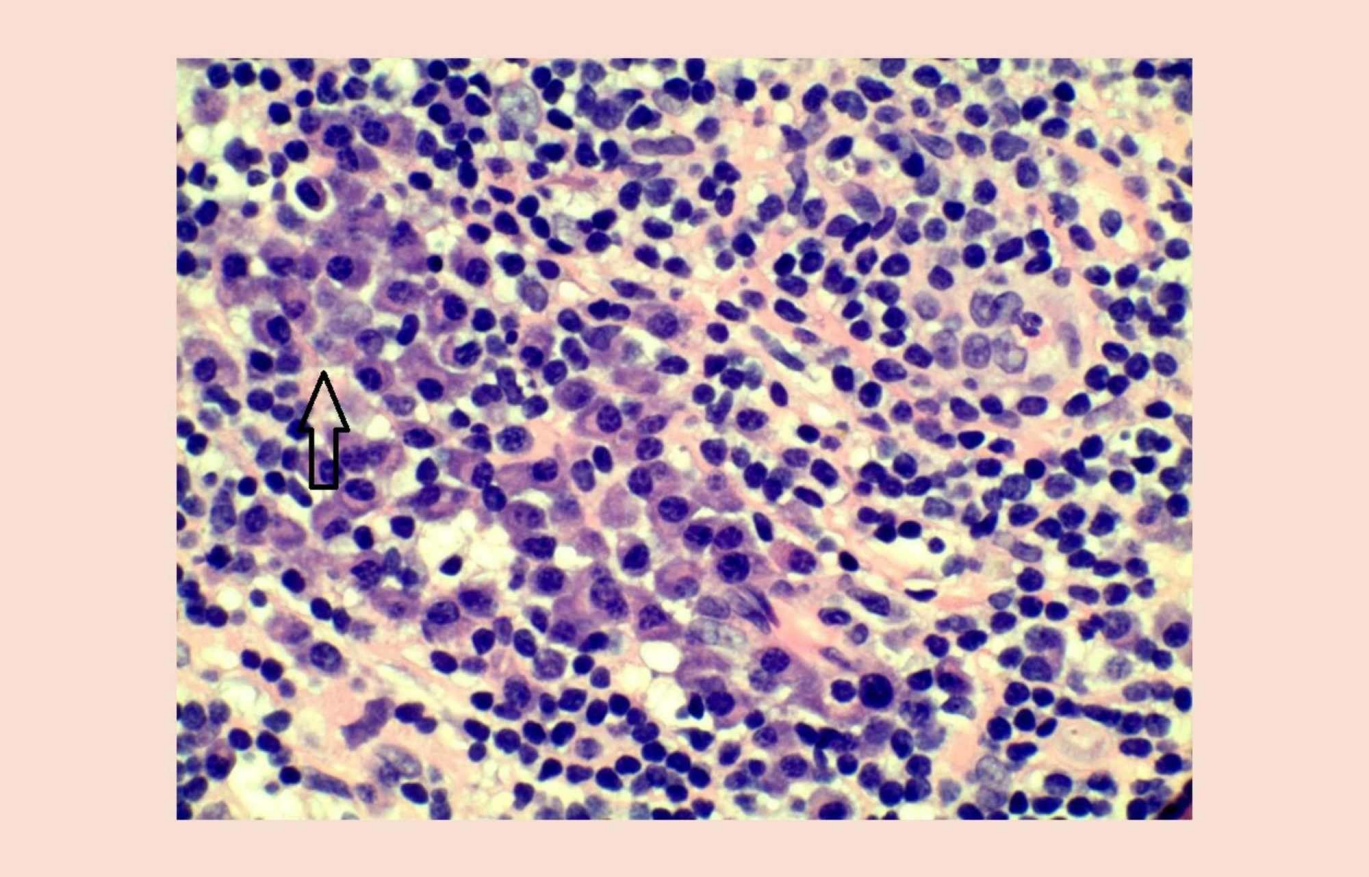
Surgical pathology demonstrated a very abnormal infiltrate with prominent histiocytes including areas with the features of extranodal sinus histiocytosis with massive lymphadenopathy.

Ultrasound (US)-guided biopsy showed proliferation of marked polytypic-appearing plasma cells associated with prominent histiocytes, with hemophagocytosis/emperipolesis and focal neutrophils. These histological characteristics were partially compatible with Rosai-Dorfman disease, especially in accordance with the presence of S100+ histiocytes. However, these findings were atypical, and the architectural pattern could not be definitively assessed due to limited biopsy sizes. In order to make a proper determination, an incisional biopsy was performed. Surgical pathology showed an abnormal cellular infiltrate with prominent histiocytes, including areas with demonstrable features of extranodal sinus histiocytosis with massive lymphadenopathy (Figure 2).

In addition, there was discernible sclerosis, signs of acute inflammation, and a benign-appearing lymphoplasmacytic component. Many of these features suggest the mass represents extranodal Rosai-Dorfman disease. On immunohistochemistry, B-raf V600E was reported negative. Modified radical neck dissection, proximal sternal resection, and superior mediastinal nodal dissection surgery was suggested. However, the patient refused the surgery and did not receive any treatment for about six months.

## Discussion

Rosai-Dorfman disease is a rare medical condition that is clinically characterized by bilateral painless cervical lymphadenopathy. Histologically, it is typified by alpha-1-anti-chymotrypsin+, S-100+, CD68+, and CD1a- histiocytes; this immunostaining pattern may help differentiate RDD from Langerhans cell histiocytosis and Erdheim-Chester disease [3]. Rosai-Dorfman disease is typically benign and self-limiting, with local excision proving to curative in most cases [4]. Treatment of Rosai-Dorfman disease is controversial; however, steroids, retinoids, cryotherapy, methotrexate, thalidomide, and/or localized radiation have been documented to show treatment efficacy in cases not amenable to surgical resection [5].

Extranodal manifestations of Rosai-Dorfman disease make up between 25-40% of cases and may present a diagnostic challenge to practicing clinicians; isolated extranodal RDD commonly mimics neoplastic entities in a variety of organ systems. The most frequent sites of extranodal involvement are the skin, bone, central nervous system, upper respiratory tract, and retro-orbital tissue [3]. Our patient experienced erosion of the sternum due to extranodal involvement of RDD in the anterior mediastinum, with encasement of the right common carotid artery and innominate vein. The prevalence of bone involvement in Rosai-Dorfman disease is approximately 5-10% of all cases; bone lesions are most commonly associated with nodal disease, unlike the case presented [6]. Blood vessel involvement by lesions attributed to RDD is exceedingly rare; several isolated cases of major vessel involvement are documented without any significant cumulative data detailing its incidence. Involvement of the pulmonary artery has been reported in only one case [7]. Kidney, liver or lower respiratory tract involvement is considered a poor prognostic factor. Major causes of mortality include amyloidosis, cytotoxicity or vital organ involvement, and can range from 2.5 to 22.5% [6, 8].

The pathogenesis of sinus histiocytosis with massive lymphadenopathy is not entirely clear. Recent studies have pointed to the role of intermediate recruiting monocytes, and alterations in their differentiation, in the development of RDD [9]. In addition to immune dysfunction, RDD may be attributed to viral etiologies, including human herpesvirus (HHV), parvovirus B19, Epstein-Barr Virus (EBV), and IgG4-associated diseases. While RDD typically manifests itself as lymphadenopathy with fever and leukocytosis, our patient was consistently afebrile with a white blood cell (WBC) count within normal limits. The most common demographic for RDD is teenage and young adult males. Less than one thousand cases have been reported since 1969. It often manifests in children and adolescents, although it has been reported up to 74 years of age [6].

Biopsy is the most important tool in the definitive diagnosis of RDD [5]. Pathology shows Rosai-Dorfman cells, which are histiocyto-macrophagic cells with positive staining for alpha-1 anti-chymotrypsin, S-100, and CD68, with negative immunostaining for CD1a [3].

Treatment plan is still controversial. Clinical monitoring is recommended if the symptoms are tolerable, as regression has been reported in many cases (20-50%) [10]. Cryotherapy, steroid, retinoid, methotrexate and thalidomide are reasonable options for cutaneous disease [5]. Chemotherapy has shown mixed results in treatment efficacy and is generally reserved for relapsed or refractory cases, in addition to initial treatment of disseminated disease [6]. In addition, surgery is often reserved for patients with vital organ involvement or extranodal disease. Given the involvement of the right carotid artery in the presented case, surgery was offered as the best alternative for this patient.

## Conclusions

This case report reviewed an unusual presentation of Rosai-Dorfman disease. Invasion of the major vessels is exceedingly rare. Treatment of Rosai-Dorfman disease is controversial. Given the involvement of the right carotid artery in the presented case, surgery was offered as the best option.
